# Enhanced recovery after surgery protocol in oesophageal cancer surgery: Systematic review and meta-analysis

**DOI:** 10.1371/journal.pone.0174382

**Published:** 2017-03-28

**Authors:** Magdalena Pisarska, Piotr Małczak, Piotr Major, Michał Wysocki, Andrzej Budzyński, Michał Pędziwiatr

**Affiliations:** 1 2^nd^ Department of General Surgery, Jagiellonian University Medical College, Krakow, Poland; 2 Department of Endoscopic, Metabolic and Soft Tissue Tumors Surgery, Kraków, Poland; 3 Centre for Research, Training and Innovation in Surgery (CERTAIN Surgery), Kraków, Poland; University Hospital Oldenburg, GERMANY

## Abstract

**Background:**

Enhanced Recovery After Surgery (ERAS) protocol are well established in many surgical disciplines, leading to decrease in morbidity and length of hospital stay. These multi-modal protocols have been also introduced to oesophageal cancer surgery. This review aimed to evaluate current literature on ERAS in oesophageal cancer surgery and conduct a meta-analysis on primary and secondary outcomes.

**Methods:**

MEDLINE, Embase, Scopus and Cochrane Library were searched for eligible studies. We analyzed data up to May 2016. Eligible studies had to contain four described ERAS protocol elements. The primary outcome was overall morbidity. Secondary outcomes included length of hospital stay, specific complications, mortality and readmissions. Random effect meta-analyses were undertaken.

**Results:**

Initial search yielded 1,064 articles. Thorough evaluation resulted in 13 eligible articles which were analyzed. A total of 2,042 patients were included in the analysis (1,058 ERAS group and 984 treated with traditional protocols). Analysis of overall morbidity as well as complication rate did not show any significant reduction. Non-surgical complications and pulmonary complications were significantly lower in the ERAS group, RR = 0.71 95% CI 0.62–0.80, *p* < 0.00001 and RR = 0.75, 95% CI 0.60–0.94, *p* = 0.01, respectively. Meta-analysis on length of stay presented significant reduction Mean difference = -3.55, 95% CI -4.41 to -2.69, *p* for effect<0.00001.

**Conclusions:**

This systematic review with a meta-analysis on ERAS in oesophageal surgery indicates a reduction of non-surgical complications and no negative influence on overall morbidity. Moreover, a reduction in the length of hospital stay was presented.

## Introduction

In the late 1990s Kehlet et al. published a series of papers on fast-track multimodal programme in colorectal surgery, which has been shown to reduce complications and shorten the length of stay (LOS) [1–3]. Subsequently, this idea evolved into a multidisciplinary instrument commonly known as Enhanced Recovery after Surgery (ERAS). This instrument integrates several perioperative elements which are now recognized as the ERAS protocol. Currently there are a number of official Guidelines published by the ERAS Society. Several meta-analyses comprising other surgical disciplines documented the benefits of ERAS [4–6]. According to Urbach et al., ERAS philosophy involves a multidisciplinary team of surgeons, anaesthetists, nurses, dieticians and physiotherapists who aim to improve the quality of care by integrating evidence-based knowledge into clinical practice [7].

So far, the evidence on the use of ERAS programmes in oesophageal surgery is sparse. There are no official ERAS guidelines and the number of papers documenting the benefits of modern multimodal perioperative care is limited. Nonetheless, it has been suggested that the majority of general principles used in gastrointestinal surgery may be applicable [8–10]. Therefore, the reports published include most of the perioperative elements widely used in other types of surgery and additionally comprise other procedure-specific items. Unfortunately, there is no unified protocol for oesophageal surgery, therefore the types and the number of items varies, depending on the surgical unit that implements the multidisciplinary perioperative protocol. Although there are several studies documenting the feasibility of ERAS in oesophageal surgery, the material on this matter is still scarce. Our study aimed to systematically evaluate and conduct a meta-analysis of the available evidence on ERAS pathways compared with traditional perioperative care patients undergoing oesophagectomy for cancer.

## Material

### Search strategy

A search was conducted by two researchers (MPe and MPi) in May 2016 of Medline, Embase, Pubmed, OVID, and the Cochrane library covering a period from January 1996 to May 2016 with language restricted to English, and using the search terms: “oesophagus”, “oesophagectomy”, “oesophageal resection”, “esophagus”, “esophagectomy”, “esophageal resection” “Ivor-Lewis” and combinations of these with: “fast track”, “enhanced recovery”, “clinical pathway”, “critical pathway”, “multimodal perioperative”, “perioperative protocol” using the Boolean operators “AND” and “OR”. Reference lists of relevant publications were assessed for additional references. Furthermore, bibliographies from other systematic reviews or meta-analyses on the subject were searched.

A paper was included when: the study concerned adult patients who underwent oesophagectomy for malignancy, the study described an enhanced recovery programme with at least four different perioperative elements and the study reported at least the overall complication rate and the length of stay. The papers included had to be either a randomized controlled trial (RCT) or a comparative study with a control group. All criteria mentioned above were required to enrol a study for further evaluation. The exclusion criteria were: the study described a single intervention in perioperative care, the study was a review, guidelines, single group studies, or the study was not in English.

Two researchers (MPi and MPe) identified and selected citations from the search independently. In the event of uncertainties relating to inclusion, a third reviewer was consulted (PiMał) until consensus was reached. Data from the included studies were further extracted independently by the two researchers. Randomized as well as nonrandomized studies were eligible as long as they met the inclusion criteria. The Jadad scale was used for the quality assessment of the RCTs, which contained randomization (0–2 points), blinding of the studies (0–2 points) and withdrawals (0–1 point). Observational studies were evaluated by the Newcastle–Ottawa Scale (NOS), which consists of three factors: patient selections, comparability of the study groups and assessment of outcomes. Missing data were obtained by contacting the authors of the respective studies. The study risk of bias was assessed using the ROBINS-I tool (Risk Of Bias In Non-randomized Studies—of Interventions) developed by the Cochrane Collaboration.

### Outcome measures

The primary outcome measure of this systematic review was overall morbidity. Secondary outcome measures were surgical complications specifying anastomotic leakage and non-surgical complications specifying pulmonary complications. Additionally, postoperative mortality, the length of hospital stay and the readmission rate were measured.

### Statistical analysis

The analysis was performed using RevMan 5.3 (freeware from the Cochrane Collaboration). Statistical heterogeneity and inconsistency were measured using Cochran’s Q tests and I2, respectively. Qualitative outcomes from individual studies were analysed to assess individual and pooled risk ratios (RR) with pertinent 95% confidence intervals (CI) favouring the ERAS treatment over non-ERAS, and by means of the Peto fixed-effects method in the presence of low or moderate statistical inconsistency (I^2^ ≤ 10%), and by means of a random-effects method (which better accommodates clinical and statistical variations) in the presence of high statistical inconsistency (I^2^ > 10%). When a study included medians and interquartile ranges, we calculated the mean ± SD using a method proposed by Hozo et. al. [11]. Weighted mean differences (WMD) with 95% CI are presented for quantitative variables using the inverse variance random-effects method. Statistical significance was observed with two-tailed 0.05 level for hypothesis and with 0.10 for heterogeneity testing, while unadjusted p-values were reported accordingly. This study was performed according to the Preferred Reporting Items for Systematic reviews (PRISMA) guidelines (S1 Table) and MOOSE consensus statement [12].

## Results

The initial reference search yielded 1,064 articles. After removing 142 duplicates, 922 articles where evaluated through titles and abstracts. This produced 53 papers suitable for full-text review. Finally, we enrolled 1 RCT and 12 comparative studies with a total of 2,042 patients (1,058 ERAS and 984 traditional protocols) (Table 1)[13–26].

**Table 1:** Study characteristics and quality assessment.

Study	Type of study	No. of patients in study/ control group	JADAD/NOS quality score	Number of ERAS elements
Blom 2013	CS	103/78	7	14
Cao 2013	CS	55/57	8	15
Findlay 2015	CS	55/77	6	11
Ford 2014	CS	75/80	7	10
Gatenby 2015	CS	27/35	6	16
Li 2012	CS	59/47	6	9
Munitiz 2010	CS	74/74	7	11
Pan 2014	CS	40/40	8	16
Preston 2013	CS	12/12	6	11
Shewale 2015	CS	386/322	6	8
Tang 2013	CS	36/27	6	10
Wang 2015	RCT	90/90	2	12
Zhao 2014	CS	34/34	8	14

**Fig 1:**
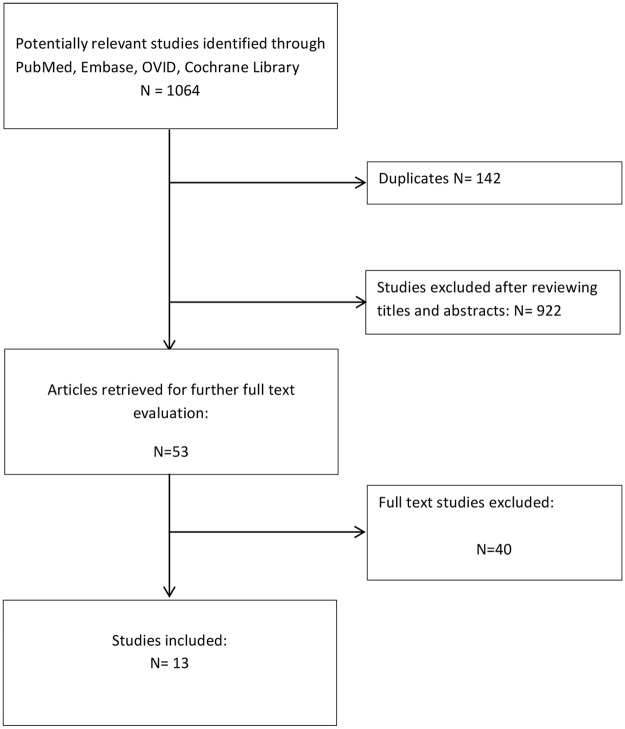
Study selection flow chart.

Author Shewale was contacted to acquire additional information regarding complications. The flowchart of the literature search and study selection is summarized in Fig 1. Protocol elements described in each study are presented in Table 2.

**Table 2:** Protocol elements.

Study	Thrombotic prophylaxis	Anti-emetics	Urinary catheter removal	Enteral feeding	Oral feeding	Goal directed fluid therapy	Discharge planning	Dietician consult	Immediate extubation	Respiratory exercise	Preoperative counselling	Preoperative nutritional support	Carbohydrate loading	Optimized anaesthesia protocols	Multi-modal analgesia	No routine use of nasogastric tubes	Removal of chest drains	Mobilization	Prevention of hypothermia
**Blom 2013**	No	Yes	No	Yes	Yes	Yes	Yes	No	Yes	No	Yes	Yes	Yes	Yes	Yes	No	Yes	Yes	Yes
**Cao 2013**	No	No	Yes	Yes	Yes	Yes	Yes	No	No	Yes	Yes	Yes	Yes	Yes	Yes	Yes	Yes	Yes	Yes
**Findlay 2015**	Yes	No	Yes	Yes	Yes	Yes	No	No	Yes	No	Yes	No	Yes	No	Yes	No	Yes	Yes	No
**Ford 2014**	No	No	Yes	Yes	Yes	No	No	Yes	Yes	Yes	Yes	No	No	No	Yes	No	Yes	Yes	No
**Gatenby 2015**	No	Yes	Yes	Yes	Yes	Yes	Yes	Yes	Yes	Yes	Yes	Yes	Yes	Yes	Yes	No	Yes	Yes	No
**Li 2012**	No	No	Yes	No	Yes	No	Yes	No	Yes	No	Yes	No	No	Yes	Yes	No	Yes	Yes	No
**Munitiz 2010**	Yes	No	Yes	No	Yes	Yes	Yes	No	Yes	Yes	No	No	No	Yes	Yes	No	Yes	Yes	No
**Pan 2014**	No	No	Yes	Yes	Yes	Yes	Yes	Yes	No	Yes	Yes	Yes	Yes	Yes	Yes	Yes	Yes	Yes	Yes
**Preston 2013**	Yes	No	Yes	Yes	Yes	Yes	Yes	Yes	Yes	No	No	No	No	No	Yes	No	Yes	Yes	No
**Shewale 2015**	No	No	Yes	Yes	No	No	Yes	No	Yes	Yes	No	No	No	No	Yes	No	Yes	Yes	No
**Tang 2013**	No	No	No	Yes	Yes	No	Yes	No	Yes	Yes	Yes	No	No	Yes	Yes	No	Yes	Yes	No
**Wang 2015**	No	Yes	No	Yes	Yes	Yes	No	No	No	No	Yes	Yes	Yes	Yes	Yes	No	Yes	Yes	Yes
**Zhao 2014**	No	No	Yes	Yes	Yes	No	Yes	No	Yes	Yes	Yes	No	Yes	Yes	Yes	Yes	Yes	Yes	Yes

### Complications

**Fig 2:**
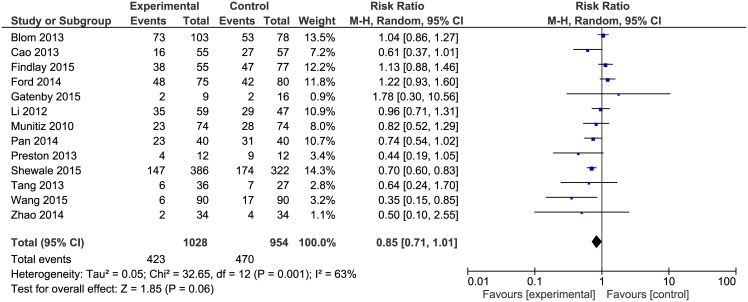
Pooled estimates of morbidity comparing enhanced recovery after surgery versus standard care. CI confidence interval, df degrees of freedom.

Overall morbidity was reported in all included papers. Complication rates were analysed with a subsequent meta-analysis (Fig 2). There were no statistically significant differences in overall complications in ERAS group (423/1028, 41%) in comparison to patients receiving traditional care (470/954, 49%): RR = 0.85, 95% CI 0.71–1.01, *p* for effect = 0.06, *p* for heterogeneity = 0.001, I^2^ = 63%.

### Surgical complications and anastomotic leakage

**Fig 3:**
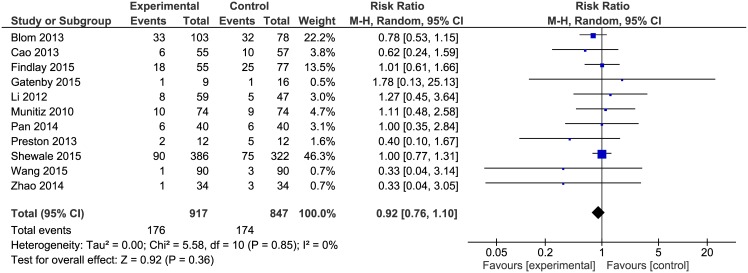
Pooled estimates of surgical complications comparing enhanced recovery after surgery versus standard care. CI confidence interval, df degrees of freedom.

Surgical complications were reported in 11 papers. The analysis (Fig 3) revealed no significant differences among the studied groups 176/917(19.2%) in ERAS group vs. 174/847(20.5%) in control group: RR = 0.92, 95% CI 0.76–1.1), *p* for effect = 0.36, *p* for heterogeneity = 0.85, I^2^ = 0%.

**Fig 4:**
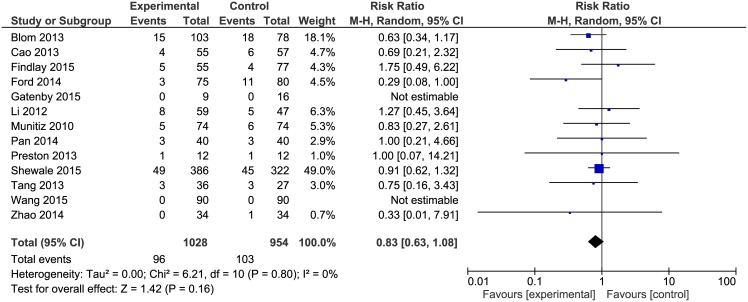
Pooled estimates of anastomotic leakage comparing enhanced recovery after surgery versus standard care. CI confidence interval, df degrees of freedom.

Anastomotic leakage was reported in all papers. The analysis (Fig 4) showed no significant variations among the studied groups 96/1028(9.3%) in ERAS group vs. 103/954(10.8%) in control group: RR = 0.83, 95% CI 0.63–1.08), *p* for effect = 0.16, *p* for heterogeneity = 0.80, I^2^ = 0%.

### Non-surgical complications and pulmonary complications

**Fig 5:**
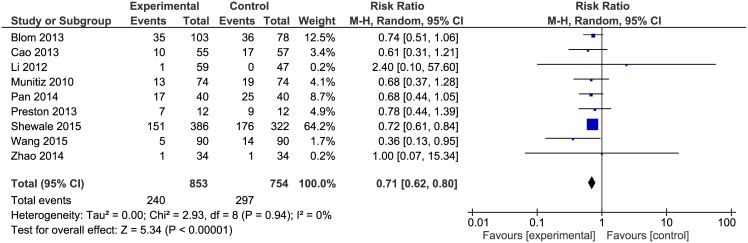
Pooled estimates of non-surgical complications comparing enhanced recovery after surgery versus standard care. CI confidence interval, df degrees of freedom.

Non-surgical complications were reported in 10 papers. Findlay et. al did not report the general number of non-surgical complications, while describing particular complications in detail. Due to the fact that some patients had multiple complications it is impossible for us to assess the number of patients with non-surgical complications, thus excluding this paper from analysis. The analysis (Fig 5) showed significant differences among the studied groups 240/853 (28.1%) in ERAS group vs. 297/754 (39.4%) in control group: RR = 0.71, 95% CI 0.62–0.80, *p* for effect < 0.00001, *p* for heterogeneity = 0.94, I^2^ = 0%.

**Fig 6:**
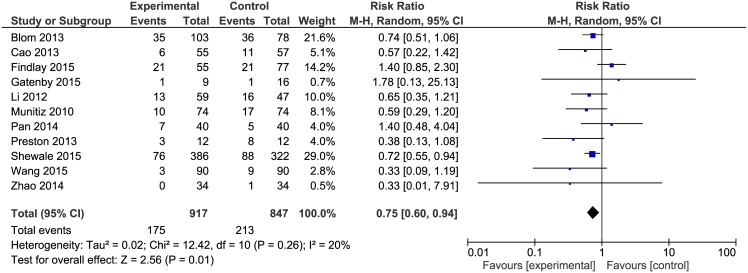
Pooled estimates of pulmonary complications comparing enhanced recovery after surgery versus standard care. CI confidence interval, df degrees of freedom.

Pulmonary complications were reported in 11 papers. The analysis (Fig 6) showed significant differences among the studied groups 175/917(19.1%) in ERAS group vs. 213/847(25.2%) in control group: RR = 0.75, 95% CI 0.60–0.94, *p* for effect = 0.01, *p* for heterogeneity = 0.26, I^2^ = 20%.

### Mortality

**Fig 7:**
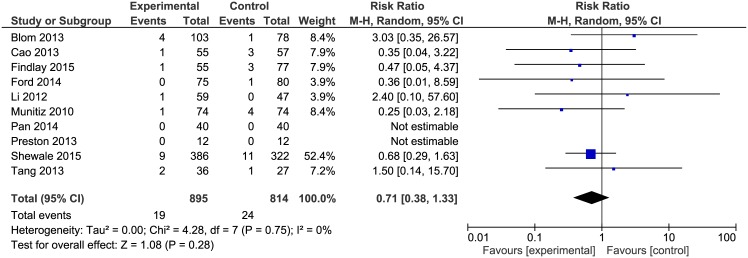
Pooled estimates of mortality comparing enhanced recovery after surgery versus standard care. CI confidence interval, df degrees of freedom.

Mortality was reported in 10 out of 13 included studies. Papers by Pan et al. and Preston et al. reported no events of mortality in their material. The analysis of mortality (Fig 7) showed no significant variations among the studied groups 19/895 (2.1%) in ERAS groups vs. 24/814 (2.9%) in control groups: RR = 0.71, 95% CI, 0.38–1.33, *p* for effect = 0.28, *p* for heterogeneity = 0.75, I^2^ = 0%.

### Length of hospital stay

**Fig 8:**
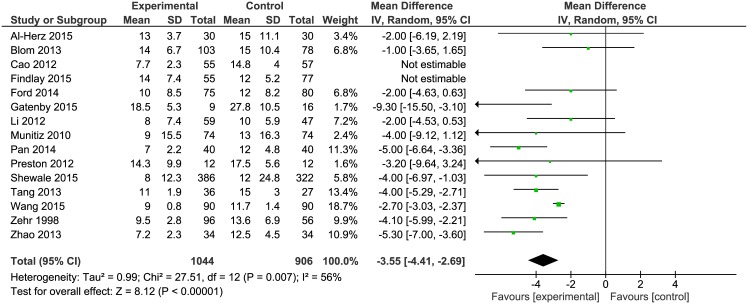
Pooled estimates of length of hospital stay comparing enhanced recovery after surgery versus standard care. CI confidence interval, df degrees of freedom.

The mean length of hospital stay (LOS) was reported in all papers and in all of them it included the primary LOS (excluding potential readmissions). When all papers were included in the analysis, a high heterogeneity (>84%) was observed. A subsequent subgroup analysis revealed 2 studies, Cao et. al and Findlay et. al, which generated most of the heterogeneity. In order to reduce heterogeneity, they were excluded from meta-analysis of this outcome. There was a significant reduction in LOS in 7 papers. The mean LOS for the ERAS group was 10.76 days while for the control group it was 14.4 days. In the study by Findlay et al. the mean LOS was longer in the ERAS group [17]. The analysis (Fig 8) showed significant differences between the studied groups: Mean difference = -3.55, 95% CI -4.41 to -2.69, *p* for effect<0.00001, *p* for heterogeneity = 0.007, I^2^ = 56%.

### 30-day readmission rate

**Fig 9:**
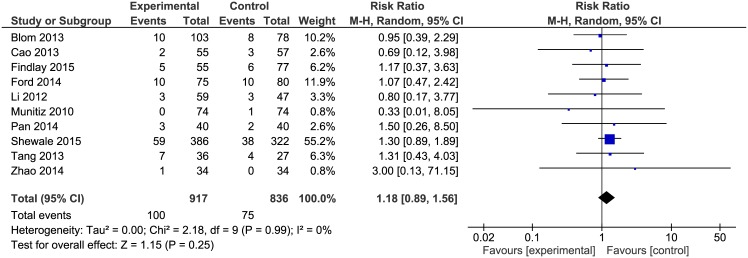
Pooled estimates of readmission rate comparing enhanced recovery after surgery versus standard care. CI confidence interval, df degrees of freedom.

Data on the readmission rate were present in 10 included articles. The analysis established no differences in the readmission rate (Fig 9) between the ERAS group 100/917 (10.9%) and the control group 75/836 (9.0%): RR = 1.18, 95% CI 0.89–1.56, *p* for effect = 0.25, *p* for heterogeneity = 0.99, I^2^ = 0%.

## Discussion

This systematic review, based on 1 RCT and 12 comparative studies enrolling 1,982 patients—1,028 in the ERAS group and 954 in the traditional care group, documents the feasibility and potential benefits of ERAS protocol in oesophageal cancer surgery. The subsequent meta-analysis of results showed a general reduction of LOS with no significant influence on surgical morbidity. Moreover, it showed a reduction in overall non-surgical and pulmonary complications. Surgical complications and anastomotic leakage rate were not affected. Also, mortality and readmission rates did not vary between groups. This suggests that multimodal modern perioperative care can be also safe in this type of surgery.

The efficacy of ERAS protocol in gastrointestinal surgery was confirmed in the previously published systematic reviews regarding colorectal, pancreatic, gastric or liver surgery [4–6,27]. These studies showed the reduction of LOS and an additional decrease in the complication rate. The number of studies with control groups regarding the application of ERAS protocol in oesophageal surgery is limited. In our literature research we came upon only one systematic review by Gemmill et al., published in 2015 [28]. This review included 11 studies, none of which was a RCT, with no subsequent meta-analysis, thus the quality of evidence was rather limited. On the contrary, our review provides a meta-analysis performed on the analysed data. However, it comprises of comparative studies and only one RCT, which obviously limits the quality of evidence.

All previous systematic reviews, regarding other surgical disciplines, showed a significant decrease in overall morbidity, mostly by decreasing non-surgical morbidity [4–6]. We did not demonstrate a reduction of overall complication rates between the groups in our review, however lack of high quality studies limits the potential evidence in this matter. Oesophageal resections are considered technically demanding, involving more than one operating field (abdominal, thoracic and cervical). Therefore, the morbidity associated with this procedure is relatively high, which was also confirmed in our review. Apart from surgical complications, high non-surgical morbidity has been reported. Interestingly, our analysis showed that standardized multimodal perioperative care may positively influence non-surgical complications. With no influence on surgical adverse events we may assume that the introduction of multimodal modern perioperative care programmes could possibly be considered as safe and beneficial. However, this statement is limited by the inconsistency of how complications were reported in the studies subject to analysis.

A common belief in the surgical world is that while ERAS shortens LOS, it inevitably leads to an increase in the readmission rate. We have shown that LOS in ERAS patients was indeed significantly shorter, but it did not affect readmissions. It was significantly shorter in 6 of 13 studies. This is may very well serve as another implication of potential safety of this type of perioperative care after oesophageal resections. Shorter LOS with non-surgical morbidity reduction and no influence on surgical complications allows us to imply that ERAS protocol improves general functional recovery. It is generally believed that modern perioperative care diminishes postoperative stress response, thus allowing faster convalescence [29]. Currently it is emphasized that a full functional recovery, rather than the postoperative hospital stay, is considered as the main goal of perioperative care [30].

The general quality of the papers included is limited. Only one analysed study was RCT with rather low quality (Jadad score 2 points), whereas the remaining were cohort studies. In order to fully assess the feasibility and potential of ERAS protocol in oesophageal surgery, more randomized trials of high quality are required.

Also, a very important aspect should be raised. Although guidelines for perioperative care in other types of surgery have been officially published by the ERAS Society, such a document for oesophageal resections does not exist. This results in a significant diversity of perioperative protocol items. While analysing the articles included we have identified at least 19 protocol elements that may be included in future guidelines. It is not surprising that none of the studies used all of these items. Their number varied between 8 and 16, which clearly demonstrates the inadequacy of the protocols described. Another issue is the interpretation of particular protocol elements in the studies included. Even though some protocols used similar items, its rendition varied, thus making it difficult to compare. For instance, chest drains were removed between postoperative day (POD) 2 and 6, patients were mobilized between POD 0 and 2, oral feeding was introduced between POD 1 and 6. In some protocols there were items which have been previously shown inappropriate or even harmful in modern perioperative care (use of nasogastric tubes, no immediate extubation, etc.). Whereas some of the programmes report using antithrombotic prophylaxis, some do not mention this element at all, yet it is difficult to comprehend that this element would not be considered conservative protocols. All of this demonstrates that it is difficult to fully assess the ERAS principles of early feeding, quick mobilization, appropriate analgesia and stress response reduction in most of the included studies. Moreover, protocol compliance was only reported by Ford et al, Li et al., Blom et al., Findlay et al. and Munitiz et al, [13,17,18,20,21]. This is important since many papers link adherence to the protocol with post-operative outcomes [31]. Due to the lack of data in other studies, it is impossible to determine the compliance rate in this review. The variability in both the number and the type of ERAS items implemented did not permit reliable subgroup analyses to identify which items might be more effective. We did not find a link between the number of the protocol elements implemented and the efficacy of the protocol. However, it has to be underlined that full evaluation may only be possible in future studies, based on unified standardised protocols or guidelines with additional information on adherence to each protocol element. Another limitation to this study is the variability of the used surgical techniques. Studies were not homogenous in this aspect and this may present a bias to our results. In most papers, types of surgery and approach used (open/minimally invasive) were usually not reported precisely. Therefore, a subgroup analysis or even a simple comparison between studies could not have been performed due to lack of necessary data. The heterogeneity of the studies, a different number of protocol elements, the lack of ERAS compliance and no unified stratification of morbidity classification prevent us from making strong conclusions about ERAS in oesophageal surgery. Although the present meta-analysis adds substantial evidence for the use of ERAS protocol in oesophageal surgery, further high quality trials are needed to fully assess its feasibility and safety.

## Conclusions

This systematic review with a meta-analysis on ERAS in oesophageal surgery indicates a reduction of non-surgical complications and no negative influence on overall morbidity. Moreover, a reduction in the length of hospital stay was presented. All analysed papers were of low quality with a high risk of bias, thus rendering limited level of evidence from these results. Therefore, further research with high quality RCTs is required to fully assess the feasibility of modern perioperative care protocols in oesophageal surgery. They may well serve as references for the forthcoming ERAS guidelines for perioperative care in oesophageal surgery.

## Supporting information

S1 Table PRISMA checklist. (DOC)
